# Electrophysiologic threshold study in air and bone conduction in children with 2 months or less age

**DOI:** 10.1016/S1808-8694(15)31074-0

**Published:** 2015-10-22

**Authors:** Silvia Nápole Fichino, Doris Ruthy Lewis, Mariana Lopes Fávero

**Affiliations:** 1 M.S. in Speech and Hearing Therapy - PUC-SP, Speech and Hearing Therapist.; 2 Pontifícia Universidade Católica de São Paulo - DERDIC/PUC-SP.; 3 PhD in Public Health - USP, Speech and Hearing Therapist.; 4 PhD in Medicine - FMUSP- Otorhinolaryngologist - DERDIC/PUCSP e do HSPM.; 5 Pontifícia Universidade Católica de São Paulo - DERDIC/PUC-SP. Mailing Address: Silvia Nápole Fichino - Avenida Jacutinga 579 apto. 142 Moema São Paulo SP 04515-030. Bolsa flexibilizada CAPES.

**Keywords:** audiology, auditory evoked response, child, early diagnosis

## Summary

The differential diagnosis of hearing loss with air and bone Auditory Brainstem Response in small children has not been enough studied in Brazil. **Aim:** To compare air and bone Auditory Brainstem Response results in children under 2 months of age with normal hearing. **Study design:** clinical with transversal cohort. **Materials and Methods:** 12 children who passed the hearing screening were evaluated using air and bone Auditory Brainstem Response. No contralateral masking was used in the bone conduction test. The responses were compared and analyzed by the McNemar test and repetitive measurements of the variance test. **Results:** There were no statistic differences between air and bone conduction Auditory Brainstem Response thresholds (p>0.05). The bone conduction latency for wave V was statistically higher than air conduction latency (p=0.000). **Conclusion:** There was agreement on the results recorded for air and bone conduction Auditory Brainstem Response for threshold intensities; latency for bone conduction wave V was statistically higher than the air conduction latency.

## INTRODUCTION

The auditory system wholeness is extremely important for human development, since hearing is the way to acquire language and speech - means through which the child organizes and understands the universe, transmits feelings, understand others, interacts with the environment and acquires knowledge.^1^

So much so that the hearing impaired may have difficulties in language development, both oral and written, in cognition and socio-emotional. In order to be possible to overcome them, enhancing communication and learning capacity. The Joint Committee on Infant Hearing (JCIH)^2^ recommends that children with hearing loss should be identified by means of a universal neonatal hearing screening (UNHS), and referred to diagnosis and intervention as early as possible.

In the United States, a study carried out in Rhode Island found a prevalence of 3.24 children with severe to profound sensorineural hearing impairment (HI) for every 1000 births.3 As for air conduction impairment, the same study showed a prevalence of 20:1000.^3^

Having seen such figures, the JCIH2 recommends that the UNHS be carried out at the newborn discharge from the hospital or in the first month of life. In cases when the screening finds a fault, the baby should be referred to an otorhinolaryngologist and a speech and hearing therapist in order to conclude the diagnosis up to the third month of life, so that therapeutic intervention may happen before the 6th month of age.

In order to confirm the diagnosis of HI, a battery of objective tests, such as acoustic immitance, transient stimulus (TSOAE) and distortion product (DPOAE) otoacoustic emissions (OAE), Brainstem Auditory Evoked Potential (BAEP), and auditory behavior that in children below 6 months of age may not correspond precisely to the toddler's hearing acuity.

The BAEP is a test that assesses the neural synchrony from an external sound stimulus, generating a complex response that represents the activity of some anatomical structures. Together with other tests, it allows us to estimate hearing, since it evaluates the auditory nerve integrity (VIII cranial nerve) all the way to the brainstem.^4^

Thus, BAEP's recording may be influenced when there is some sound conduction impairment (sensorineural or conductive hearing loss), or some change in neural conduction (e.g. some auditory neuropathy or a tumor).^5^,^6^,^7^

The BAEP's triggering stimulus, usually a click, may be given by the air conduction (AC), which is usually carried out, or by bone conduction (BC), by means of a bone vibrator placed on the postero-superior auricular portion at 45° from the external acoustic meatus orifice.^8^

In those cases in which the AC BAEP is altered in newborns, it is recommended to do a BC BAEP^2^,^4^,^7^,^9^, for the prevalence of conductive hearing loss in this population, as mentioned before, as for the diagnosis difficulty in this age range. In such cases, when we compare the results, we see the BC BAEP threshold within normal ranges^9^, ^10^, ^11^, ^12^ and the AC BAEP threshold is increased.

Nonetheless, there are very few research papers using BC BAEP, and the literature shows much protocol disputes, making it difficult to classify a result as normal, its comparison with AC results and, consequently, the clinical applicability of this method. Thus, the goal of the present investigation was to compare AC and BC BAEP responses in children up to 2 months of age without hearing loss.

## MATERIALS AND METHODS

This investigation was carried out in the department of electrophysiology of our institution, from March to April of 2004. The project was approved by the Ethics Committee of our University, under protocol # 0142/2003 and by its Research Committee.

We evaluated twelve children with mean age of 20 days (standard deviation of 7.89 days) from the neonatal hearing screening service, whose parents accepted participating in this study and signed an informed consent form.

Inclusion criteria were:

• no complaints regarding the children's hearing;
• no pre, peri and/or post-natal complications, or risk factors for hearing impairment according to the JCIH2;
• type “A” tympanometry, with a compliance peak around 0daPa, of which variation would not exceed −100 daPa (GSI 33 immitancemeter with a 226 Hz probe);
• presence of transient stimulus otoacoustic emissions (TSOAE), with general reproducibility^3^ 50% and with at least the 3 last frequency bands with a noise-signal ratio of 6 dBpSPL and probe sound stability^3^ 75% (ILO292 - Otodynamics);
• attention reaction to sound and cochleo-eyelid reflex for the reco-reco and agogô instruments, respectively;
• presence of waves I, III and V, with absolute interpeak and latency times within normal ranges for the age during BAEP exam at 80 dBHL (Smart EP - Intelligent Hearing Systems);

The children who did not have the aforementioned criteria were referred to otorhinolaryngological and speech and hearing assessment.

We recorded BAEPs waves by AC and BC using the version 2.1X. Smart EP - Intelligent Hearing Systems device, with the children under natural sleep, and usually after a meal.

The reference leads were deployed on the right (A2) and (A1) mastoid bones, and the live (Fz) and ground (Fpz) electrodes were placed on the forehead, after proper skin cleaning and the impedance between electrodes was considered less than 5000 ohms.

In order to record BAEPs waves by AC we used EARTONE 3^o^ insertion phones, with proper fitting for newborns. We survey waves I, III and V in the intensities of 80 dBHL, 60 dBHL, 40 dBHL and 30 dBHL.

For BC BAEP recording we used a Radioear B-71 bone vibrator deployed on the postero-superior ear portion, fixing it with a 1582 model, 5cm wide, autoadherent 3M Coban elastic band, with power of 400 ± 25g, measured by a model 8264-M scaled Ohaus - Spring Scale. Wave V was investigated in the intensities of 40dBHL and 30 dBHL. The test was carried out without contralateral masking.

The parameters used for BAEPs recordings are depicted on Chart 1.

**Chart 1:** Parameters used in BAEP recordings by AC and BC; adapted from Hood.4

Parameter	Air conduction	Bone conduction
Stimulus	100msec click	100msec click
Polarity	Alternated	Alternated
Intensities	80,60,40,30 dB	40,30 dB
Stimulus frequency	27.7/Sec	27.7/Sec
Window	25msec	25msec
Filters	100-3000 Hz	100-3000Hz
Number of stimulus	At least 2000	At least 2000
Reproductions	2 records	2 records

In order to compare the results attained by Ac and BC, we used:

1-presence or absence of wave V by BC in the intensities of 40 and 30 dBHL with or without wave V by AC in the right and left ear of each participant (95% confidence interval) on the following way:

40 dBHL: wave V VO x wave V VA RE

40dBHL: wave V VO x wave V VA LE

30 dBHL: wave V VO x wave V VA RE

30 dBHL: wave V VO x wave V VA LE

2-BC wave V latencies mean values with AC wave V latency mean values on both, the right and the left ears in the intensities of 40 and 30 dBHL.

The first association was tested by the McNemar test and the second by the variance analysis with repetitive measures, according to aforedescribed methods.^13^ For both, we considered the statistical significance level of p£ 0.05.

## RESULTS

At 40 dBHL all the assessed children (100%) responded both by air and bone conduction; and 11 children (92%) responded by air and bone conduction on the right side. At 30 dBHL 75% and 58% of the children presented response both for the AC and BC on the right and left ears, respectively. (Tables 1, 2 and 3)

**Table 1:** Joint frequencies and percentage distribution of AC and BC response occurrence, right ear, intensity of 40 dBHL.

		VA LE 30 - V		Total
VO - 30 - V		No	Yes	
No	Frequency	1	1	2
	%	8%	8%	17%
Yes	Frequency	1	9	10
	%	8%	75%	83%
Total	Frequency	2	10	12
	%	17%	83%	100%

**Table 2:** Joint frequencies and percentage distribution of AC and BC response occurrence, right ear, intensity of 30 dBHL.

		VA RE 30 - V		Total
VO - 30 - V		No	Yes	
No	Frequency		2	2
	%		17%	17%
Yes	Frequency	3	7	10
	%	25%	58%	83%
Total	Frequency	3	9	12
	%	25%	75%	100%

**Table 3:** Joint frequencies and percentage distribution of AC and BC response occurrence, left ear, intensity of 30 dBHL.

		VA LE 30 - V		Total
VO - 30 - V		No	Yes	
No	Frequency	1	1	2
	%	8%	8%	17%
Yes	Frequency	1	9	10
	%	8%	75%	83%
Total	Frequency	2	10	12
	%	17%	83%	100%

Tables 4 and 5 present the response occurrence ratios for each ear in AC and BC and at each intensity with their respective confidence intervals and p values. We noticed that there were no statistical differences in responses between the two paths (p>0.05).

**Table 4:** Response estimate likelihood at the intensity of 40 dBHL

Situation	N	p	Confidence interval
AC RE 40 V	12	0,92	(0,62; 1,0)
AC LE 40 V	12	1	(0,78; 1,0)
BC VO 40 V	12	1	(0,78; 1,0)

**Table 5:** Response estimate likelihood at the intensity of 30 dBHL

Situation	N	p	Confidence interval
AC RE 30 V	12	0,75	(0,43; 0,95)
AC LE 30 V	12	0,83	(0,52; 0,98)
BC 30 V	12	0,83	(0,52; 0,98)

As to AC wave V latency time, at 40 dBHL, we recorded a mean time of 7.39ms, with a minimum of 6.35ms and a maximum of 8.6ms. And, at 30 dBHL, by AC, we recorded a mean time of 7.94ms, with a minimum of 6.75ms and maximum of 9.7ms.

As to BC, at 40 dBHL, we recorded a mean time of 9.18ms, with a minimum of 8.45ms and maximum of 9.55ms. And, at 30 dBHL, by BC, we recorded a mean time of 9.72ms; 9.05ms the minimum and 10.7ms the maximum time recorded.

**Figure 1:**
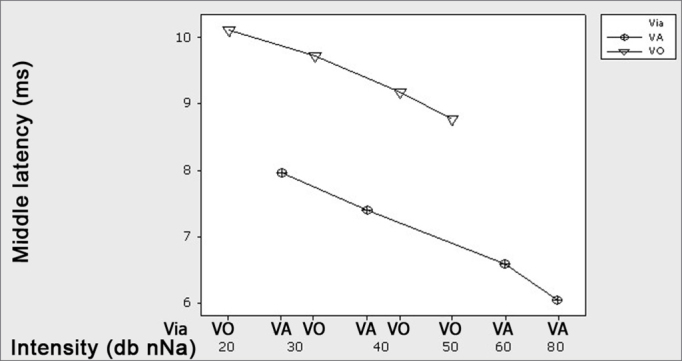
Graph, showing the mean latencies (ms) for wave V at each intensity level (dBHL) for Air and Bone conduction – -o- AC -<- BC.

Figure 1 shows the latency times mean values found by AC and BC.

## DISCUSSION

BC BAEP, although it is recorded and interpreted as its AC counterpart, does bear some peculiarities. In executing this protocol, we had some difficulties that should be stated for future investigations.

The bone vibrator emits electromagnetic energy, that interferes in the recording, causing artfacts.^4^,^9^,^14^, ^15^, ^16^ In order to minimize these artifacts, the vibrator should be placed as much away as possible from the lead, the latter should be placed on the earlobe or the auditory canal, or even use alternate polarity stimuli.^9^ In the present investigation we used alternate polarity, but we were unable to fit the lead to the earlobe, keeping it in the postero-superior ear region.

These electromagnetic artifacts make it difficult to visualize waves I and III, and because of it, we chose to study only the wave V. Moreover, the maximum intensity emitted by the bone vibrator is of, approximately, 50 dBHL, and this generates a small response amplitude^9^,^10^,^14^ making it even more difficult to identify the more distal waves. This limited dynamic wave makes it difficult to make a differential diagnosis of severe/profound sensorineural hearing loss from a severe/profound mixed hearing loss.^14^

Both the position and the power of the bone vibrator are able to alter wave V latency time.^15^ For this reason, the bone vibrator must be always used in the same position and at the same power level in all the subjects; if not, the test may yield a long latency time, altered when compared to the standard. That is why we used a scale as a means to keep a constant compression force on the elastic band that holds the bone vibrator.

There is also the issue of masking the contralateral ear. The click interaural attenuation by bone conduction in children below 1 year is of approximately, 25 to 35 dBHL, needed mainly for stronger intensities, of masking the non-tested ear.^14^ Thus, in intensities up to 35 dBHL it is not necessary to use contralateral masking when we test neonates and small children.^14^ They also mention masking difficulties in small children, for example, in the cases of those sleeping over the non-tested ear, since they may easily awake with its manipulation, and also in cases of bilateral conductive hearing loss. ^14^

In this first study, because of the children's ages, the presence of otoacoustic emissions during hearing screening (inclusion criteria) and, still, because at the time we had no practical experience with BC BAEP, we chose not to use contralateral masking. Notwithstanding, we believe in the need and relevance of BC BAEP with contralateral masking for later clinical application, since there may be unilateral hearing loss with screening failure on this side and masking is the only option we have in order to isolate the ears and have reliable results for the right and left ear separately.

Comparing the presence of waves V obtained by AC and BC in the intensities near the auditory threshold, we did not obtain statistically significant differences, indicating that there is a response agreement for BAEPs captured by both pathways in normal children, and further suggesting that a difference between both traces indicates conductive hearing loss. Moreover, analyzing results from Table 5, we see that, if VO response is used as a normality criterion at 30 dBHL, the likelihood of having wrongly classified a child with normal hearing is of 0.17 (1-0.83).

These data corroborate those from other investigations^16^,^17^, suggesting that the electrophysiologic threshold difference recorded by AC and BC (gap) may indicate the conductive component magnitude, as we have with behavioral audiometry.

As to wave V latency time, comparing the mean values from the recording obtained by air conduction with bone conduction wave V recordings in the intensities of 40 and 30 dBHL, we obtained latency values statistically higher in BC when compared to AC (p=0.000), (Figure 1), regardless of the intensity tested (p = 0.856). Many authors report that BC recorded latency time is higher that that of AC15-18, and this may happen because of the difference in the energy transmission by the transducers (phones and bone vibrator)19 and click frequency spectrum by bone conduction; besides the bone vibrator power and positioning.^14^, ^15^, ^16^, ^17^, ^18^

As to the click stimulus frequency range by AC and BC, some authors^16^,^18^ studied AC and BC stimuli and observed that at the range recorded by BC there is a frequency peak at 1-2kHz while by AC, this peak is between 2-4kHz. Thus, cochlea stimulation occurs differently because of the transducers17, and by BC there is stimulation of the middle portion towards the cochlear apex, in other words, a longer transmission time through the basal membrane, differently from the AC stimulation, which hits the cochlear base.^16^,^18^ Thus, BC recording occurs after the AC response.

Now, as to the bone vibrator power and positioning, studies15 show that the weaker the bone vibrator placement, the larger will be the latency time. In the present investigation, we used a strength of 400 ± 25g and, thus, for future comparisons we should use the same protocol. We know that, if we increase the power with which the bone vibrator is tied to the skull, we reduce the latency time recorded.15 The authors have shown that, when they used powers of 425g, 325g or 225g, the BC latency time was higher than that of AC. However, when they used a power of 525g the opposite happened, in other words, the AC latency time was greater.15 The authors suggest using a power of 425 or 525g since lower power reflects less effectiveness in cochlear stimulation, and there is also the possibility that the bone vibrator may shift with the child's movements. ^14^,^15^

In the present investigation, we kept constant both, the bone vibrator power and positioning, keeping it with elastic bands, and we did not have accidental shifting and alterations in the findings.

Some authors we consulted^4^,^15^, ^16^, ^17^, ^18^ suggest that, before putting the BAEP to clinical use by AC and BC, the clinician should standardize the equipment and the protocol to be used, testing both, children and adults, checking if his/her findings are in agreement with those in the literature, thus establishing criteria for normality for AC and BC BAEP in his/her service. Thus, he/she may compare the clinical findings with the normal values established and, should a gap occur between the AC and BC values, classify the hearing loss as sensorineural, conductive or mixed.

## CONCLUSIONS

By comparing the BAEP's responses by AC and BC in children up to 2 months of age without hearing loss, we may conclude that:

1) There are no statistically significant differences as to the presence of wave V by AC and BC in the intensities close to the auditory threshold.
2) Wave V latency registered by BC is statistically higher than the latency recorded by AC.
